# Allergic Asthmatics Show Divergent Lipid Mediator Profiles from Healthy Controls Both at Baseline and following Birch Pollen Provocation

**DOI:** 10.1371/journal.pone.0033780

**Published:** 2012-03-15

**Authors:** Susanna L. Lundström, Jun Yang, Henrik J. Källberg, Sarah Thunberg, Guro Gafvelin, Jesper Z. Haeggström, Reidar Grönneberg, Johan Grunewald, Marianne van Hage, Bruce D. Hammock, Anders Eklund, Åsa M. Wheelock, Craig E. Wheelock

**Affiliations:** 1 Division of Physiological Chemistry II, Department of Medical Biochemistry and Biophysics, Karolinska Institutet, Stockholm, Sweden; 2 Department of Entomology and Cancer Center, University of California Davis, Davis, California, United States of America; 3 Institute of Environmental Medicine, Karolinska Institutet, Stockholm, Sweden; 4 Clinical Immunology and Allergy Unit, Department of Medicine, Karolinska Institutet, Stockholm, Sweden; 5 Division of Respiratory Medicine, Department of Medicine, Karolinska Institutet, Stockholm, Sweden; Leiden University Medical Center, The Netherlands

## Abstract

**Background:**

Asthma is a respiratory tract disorder characterized by airway hyper-reactivity and chronic inflammation. Allergic asthma is associated with the production of allergen-specific IgE and expansion of allergen-specific T-cell populations. Progression of allergic inflammation is driven by T-helper type 2 (Th2) mediators and is associated with alterations in the levels of lipid mediators.

**Objectives:**

Responses of the respiratory system to birch allergen provocation in allergic asthmatics were investigated. Eicosanoids and other oxylipins were quantified in the bronchoalveolar lumen to provide a measure of shifts in lipid mediators associated with allergen challenge in allergic asthmatics.

**Methods:**

Eighty-seven lipid mediators representing the cyclooxygenase (COX), lipoxygenase (LOX) and cytochrome P450 (CYP) metabolic pathways were screened via LC-MS/MS following off-line extraction of bronchoalveolar lavage fluid (BALF). Multivariate statistics using OPLS were employed to interrogate acquired oxylipin data in combination with immunological markers.

**Results:**

Thirty-two oxylipins were quantified, with baseline asthmatics possessing a different oxylipin profile relative to healthy individuals that became more distinct following allergen provocation. The most prominent differences included 15-LOX-derived ω-3 and ω-6 oxylipins. Shared-and-Unique-Structures (SUS)-plot modeling showed a correlation (R^2^ = 0.7) between OPLS models for baseline asthmatics (R^2^Y[cum] = 0.87, Q^2^[cum] = 0.51) and allergen-provoked asthmatics (R^2^Y[cum] = 0.95, Q^2^[cum] = 0.73), with the majority of quantified lipid mediators and cytokines contributing equally to both groups. Unique structures for allergen provocation included leukotrienes (LTB_4_ and 6-*trans*-LTB_4_), CYP-derivatives of linoleic acid (epoxides/diols), and IL-10.

**Conclusions:**

Differences in asthmatic relative to healthy profiles suggest a role for 15-LOX products of both ω-6 and ω-3 origin in allergic inflammation. Prominent differences at baseline levels indicate that non-symptomatic asthmatics are subject to an underlying inflammatory condition not observed with other traditional mediators. Results suggest that oxylipin profiling may provide a sensitive means of characterizing low-level inflammation and that even individuals with mild disease display distinct phenotypic profiles, which may have clinical ramifications for disease.

## Introduction

Asthma is a respiratory tract disorder with global implications that is characterized by airway hyper-responsiveness and chronic airway inflammation [1], [2], [3], [4]. The disease is frequently associated with increased IgE-mediated sensitization and correlates with other allergic diseases including eczema and rhinitis [5], [6]. Allergic asthma is characterized by allergen-specific IgE antibody production and secretion of Th2 cytokines (*e.g.*, IL-4 and IL-13) by T-helper cells with subsequent activation of mast cells, eosinophil infiltration and airway smooth muscle constriction [7], [8], [9], [10]. Lipid mediators in the form of oxidized fatty acids (oxylipins) are known markers of early asthmatic and anaphylactic patient responses to allergens [11], [12], [13], [14]. Oxylipins are biosynthesized from unsaturated fatty acids via three main pathways: lipoxygenase (LOX), cyclooxygenase (COX) and cytochrome P450 (CYP) [15], [16]. Oxylipins include the well-known eicosanoids synthesized from arachidonic acid (AA; *e.g.*, leukotrienes and prostaglandins), as well as mediators synthesized from other related ω-6 and ω-3 fatty acids (fatty acids containing the initial double bond 6 or 3 carbons respectively from the terminal methyl group of the fatty acid backbone) [17], [18].

The roles of specific oxylipins in the etiology and pathology of asthma have been extensively reported [19], [20], [21], [22], [23], [24], [25]. In particular, oxylipins regulated by the COX and LOX pathways have been shown to be elevated, both at baseline levels and/or following provocation, in allergic asthma [13], [14], [26], [27], [28]. For example, in asthmatics the cysteinyl-leukotrienes exert a causative role in allergen-induced airway inflammation through selective increase in eosinophils in the airways [22], and prostaglandin as well as the 15-LOX product 15-hydroxyeicosatetraenoic acid (15-HETE) production increases following allergen exposure [29]. 15-LOX, in particular is significantly elevated in epithelial cells in the airways of asthmatic subjects [30], [31], [32] and regulated by the cytokines IL-4 and IL-13 [33], [34]. 15-LOX activity has been hypothesized to have both pro-inflammatory and anti-inflammatory characteristics in the lung [31], [32], [35], [36]. For instance, the primary 15-LOX product formed from AA, 15-hydroperoxyeicosatetraenoic acid (HpETE), can be dehydrated to pro-inflammatory 14,15-leukotriene derivatives (eoxins) [37], [38], or serve as the precursor to the anti-inflammatory lipoxins [39]. A protective function for 15-LOX has also been demonstrated by its role in the formation of ω-3-derived resolvins and protectins [17], [40]. Accordingly, oxylipin biology is a complex interaction of pro- and anti-inflammatory signals that are dependent upon the temporal sequence of production, and should be examined within the context of the complex milieu of resident pulmonary cells and infiltrating inflammatory cells as well as interactions with other nonlipid inflammatory mediators. In addition, dietary shifts in fatty acid consumption can affect oxylipin levels and speciation [41].

In this study, we quantified oxylipins in bronchoalveolar lavage fluid (BALF) from mild allergic asthmatics 24 h following birch pollen provocation compared to baseline levels in both asthmatic and healthy control subjects. An oxylipin metabolic profiling approach was applied in which a broad selection of compounds were quantified representing major components of the relevant biological pathways. Multivariate statistics were used to correlate oxylipin levels with clinical parameters, cytokines, and data from analysis of cells and inflammation markers by fluorescence activating cell sorting (FACS). This approach demonstrates the utility of applying a broad scale metabolic profiling method to investigate mechanisms in asthma, and particularly the integrative power of combining analytical data with patient clinical information using multivariate statistics.

## Methods

### Clinical data and study design

Detailed descriptions of the subjects and study design are provided elsewhere [42]. Briefly, 8 birch pollen allergic patients with mild asthma (age 22–42 years, 4 females) and 10 non-allergic healthy control subjects (age 22–37 years, 5 females) were included in the present study (Table 1). All asthmatic patients had allergen-specific IgE to inhaled birch pollen (>2 kU/l), while non-allergic controls were negative (<0.35 kU/l) to Phadiatop® (a mix of common inhalant allergens, Immuno CAP System, Phadia AB, Uppsala, Sweden). Allergen challenge was performed by inhalation using doses required for 20% drop in FEV_1_ as shown in Table 1. Asthmatic subjects were asymptomatic, with stable mild intermittent disease, with occasional use of inhaled short-acting β2-agonists. The wash out period was a minimum of 4 weeks for inhaled steroid use following exacerbations, and a 2 week minimum for anti-histamines, anti-leukotrienes and non-steroidal anti-inflammatory drugs (NSAIDs). Exacerbations and airway infections were prohibited during the last 4 weeks prior to sampling. The study was performed outside of birch pollen season. Sampling by bronchoalveolar lavage (BAL) was carried out as described previously [43] on all subjects at 1) unprovoked baseline levels and 2) a second time point within 3 months, and with at least 14 days between the two time points, on the asthmatic subjects following bronchoprovocation with birch pollen extract (Aquagen SQ, ALK, Copenhagen, Denmark), corresponding to a final drop in FEV_1_≥20% from the postdiluent baseline value [44]. BAL was carried out 24 h post-challenge. BALF was strained through a Dacron net (Millipore, Bedford, Ireland), centrifuged at 400 *g* for 10 min at 4°C and kept at −80°C until use. All participants gave their informed written consent and the study was approved by the Stockholm Regional Ethics Committee (Case number 2005/1259-31/2).

**Table 1:** Clinical data of participating subjects.

Subject^a^	Diagnosis^b^	Age^c^	Gender^d^	FEV_1_%^e^	IgE birch^f^
1	A	42	F	98	8
3	A	36	F	101	31
4	A	22	M	88	20
6	A	32	F	104	5
7	A	34	F	101	3
8	A	25	M	102	54
12	A	24	M	82	16
13	A	24	M	103	100
4	H	37	M	117	N/A
5	H	25	M	120	N/A
6	H	25	F	96	N/A
7^g^	H	26	F	97	N/A
8^g^	H	26	F	111	N/A
9^h^	H	22	M	99	N/A
10	H	25	M	99	N/A
11	H	23	F	99	N/A
12	H	24	F	99	N/A
13	H	26	M	105	N/A

a Subject numbering is presented as originally published by Thunberg *et al.* [42].

b Asthma (A), Healthy (H).

c Age in years at time of study inclusion.

d Female (F), Male (M).

e FEV_1_% values at baseline. A Student's t-test of the groups indicated that there was no different between the two populations (p = 0.11).

f kU/l required for 20% drop in FEV_1_%, as reported by Thunberg *et al.* [42].

g Oxylipin profiling of subject 7 and 8 was performed in a pooled sample.

h Individual 9 was incorrectly reported by Thunberg *et al.* [42] and is a 22 year old male healthy individual.

### Oxylipin extraction and analysis

Analytical oxylipin standards, deuterated oxylipin internal standards and the technical standard *N*-cyclohexyl-*N*′-dodecanoic acid urea (CUDA, also termed a Type II internal standard [45]) were obtained from Cayman Chemical (Ann Arbor, MI, USA), Larodan Fine Chemicals AB (Malmö, Sweden), Enzo Life Sciences (Farmingdale, NY, USA) or synthesized in-house [45]. A list of all standards is provided in the Supporting Information (Table S1). Off-line solid-phase extraction (SPE) was performed using Waters Oasis HBL 60 mg cartridge columns (Milford, MA, USA) as previously described [45], [46], [47]. A detailed description of the instrument method is given elsewhere [45]. An Agilent 1200 SL separation module (Santa Clara, CA, USA) coupled to ABI 4000 QTRAP® hybrid triple quadrupole/linear ion trap mass spectrometer (Foster City, CA, USA) was used for analyses and separation was performed via a 2.1×150 mm Eclipse plus C18 column with a 1.8 µm particle size (Agilent, Santa Clara, CA, USA). Oxylipins were quantified using internal standard methods as previously described [45]. A sample chromatogram for the full oxylipin metabolic profiling method is shown in Figure S1A. In addition, Figure S1B displays the separation of 6-*trans*-LTB_4_ and 5,6-DiHETE from other purported isomers. However, due to a lack of analytical standards, these other compounds were not definitively identified or quantified and therefore are not reported in either this study or previous publications [45], [46], [47]. Oxylipins detected above the limit of quantification (LOQ) were quantified, and the values shown in Table 2 are normalized by the BALF recovery volume (V_(Recovered volume)_/V_(Instilled volume)_) [47]. Concentrations not adjusted for BALF recovery as well as BALF recovery information are provided in Table S2 and Table S3, respectively. The long-term stability of the full profile of oxylipins in BALF has not been evaluated, but a recent study evaluated stability in a range of conditions in cell culture [48].

**Table 2:** Oxylipin concentrations in BALF from 1) Healthy controls 2) Asthmatic controls and 3) Asthmatics following provocation.

PUFA^a^	Oxylipin^b^ (pM)	Healthy controls (HC)		Asthmatic controls (AC)		Asthmatics following provocation (AFP)		Significance^c^			
								HC/AC	HC/AFP	AC/AFP	Trend^d^
		AV^e^	CV^f^	AV	CV	AV	CV	p	p	p	p
AA^g^	5-HETE	89	66	135	62	131	62	0.20	0.22	0.83	0.071
	12-HETE	32	69	80	51	104	58	*0.0081*	*0.012*	0.30	*0.022*
	15-HETE	337	73	734	66	991	63	*0.046*	*0.022*	0.066	0.091
	15-KETE	110	64	252	76	294	62	0.080	*0.026*	0.28	0.091
	5,6-DiHETE	2.6	89	3.1	65	3.7	74	0.68	0.42	0.48	*0.030*
	5,15-DiHETE	5.4	98	11	65	12	57	0.085	*0.048*	0.79	*0.028*
	11,12-DiHETrE	0.9	82	1.4	41	1.3	74	0.12	0.38	0.62	*0.045*
	14,15-DiHETrE	2.9	50	3.6	20	3.3	35	0.24	0.57	0.56	0.070
	11(12)-EpETrE	3.2	44	7.6	92	4.0	59	0.12	0.42	0.22	0.39
	LTB_4_	53	71	72	59	81	53	0.35	0.17	0.44	*0.020*
	6-trans-LTB_4_	22	56	32	58	39	45	0.18	*0.030*	0.051	*0.030*
	TXB_2_	41	65	47	52	78	119	0.66	0.30	0.36	0.20
	PGE_2_	5.1	64	6.1	33	9.8	56	0.44	*0.046*	0.16	*0.040*
	PGD_2_	3.4	84	5.6	75	7.7	89	0.23	0.11	0.52	*0.020*
LA^h^	9-HODE	201	66	224	55	190	63	0.73	0.85	0.63	0.20
	9-KODE	129	73	197	77	138	68	0.27	0.84	0.21	0.37
	13-HODE	785	74	1250	60	1530	51	0.17	*0.039*	0.15	*0.022*
	EKODE	244	65	541	54	449	55	*0.018*	0.057	0.41	*0.0060*
	9,10,13-TriHOME	68	43	122	61	104	25	0.087	*0.016*	0.52	*0.022*
	9,12,13-TriHOME	279	46	537	54	516	27	*0.043*	*0.0024*	0.85	*0.0060*
	9,10-DiHOME	221	53	440	69	463	57	0.090	*0.039*	0.82	*0.0030*
	9(10)-EpOME	914	27	1370	49	1570	42	0.10	*0.026*	0.42	0.060
	12,13-DiHOME	235	52	460	74	467	60	0.11	0.057	0.95	0.060
	12(13)-EpOME	1000	30	1540	48	1770	38	0.086	0.16	0.39	0.060
DGLA^i^	15-HETrE	38	63	81	59	116	71	*0.032*	*0.031*	0.18	0.091
α-LA^j^	9-HOTE	8.0	63	9.4	44	6.8	41	0.56	0.54	0.16	0.41
	13-HOTE	33	70	67	72	76	48	0.083	*0.011*	0.45	*0.022*
	12(13)-EpODE	2.3	61	4.3	55	5.0	48	*0.048*	*0.010*	0.41	*0.0030*
EPA^k^	5-HEPE	8.2	86	13	54	13	65	0.15	0.24	0.68	0.080
	12-HEPE	6.2	52	16	58	17	38	*0.021*	*0.00051*	0.71	*0.0030*
	15-HEPE	32	70	99	70	110	53	*0.030*	*0.0064*	0.27	0.091
DHA^l^	17-HDoHE	162	65	452	76	517	60	0.051	*0.014*	0.59	*0.0030*
15-LOX^m^	ω-6 (n = 10)^n^	1980	52	3490	46	4000	39	*0.034*	*0.0064*	0.29	*0.022*
	ω-3 (n = 5)	242	60	644	70	727	53	*0.040*	*0.0088*	0.55	*0.022*
	Total 15-LOX (n = 15)	2230	52	4130	47	4720	41	*0.025*	*0.0049*	0.31	*0.022*

a Polyunsaturated fatty acid.

b Oxylipin levels are reported as concentration (pM) and were normalized to the BAL volume recoveries. A complete list of all oxylipin acronyms is provided in Table S1.

c Statistical significance was calculated with either an unpaired or paired Student's t-test. Values with p<0.05 are shown in italics with two significant figures.

d The median concentration (pM) among the healthy controls (HC) regarding each oxylipin was used as a cut off limit for all three groups (*i.e.*, Healthy Controls, Asthmatic Controls and Asthmatics Following Provocation), with values provided in Table S8. All p-values regarding trend are one-sided.

e Average,

f Coefficient of variance,

g Arachidonic acid,

h Linoleic Acid,

i Dihomo-γ-linolenic acid,

j α-Linolenic acid,

k Eicosapentaenoic acid,

l Docosahexaenoic acid.

m 15-Lipoxygenase products.

n The numbers in parentheses indicate the number of oxylipin species analyzed for each subgroup.

### Immunological markers

Bronchoalveolar lavage cells were cytospun and stained with May-Grünwald-Giemsa, followed by differential counting of macrophages, lymphocytes, eosinophils, neutophils and mast cells. Cytokine levels in BALF were measured by cytometric bead array analyses as previously reported [42]. Surface- and intracellular staining of BAL cells followed by flow cytometry analyses of a number of markers for common immune cells, as well as activation/regulatory markers, were performed as earlier described [42]. Four panels of FACS antibodies were used for the analysis as described in Table S4. Data for some of the markers have been published earlier [42]. The data used for multivariate analysis and values presented in Table 3 represent percentage of expressed markers in defined cell populations as listed in Table S5.

**Table 3:** Cytokine and BAL cell levels in BALF from 1) Healthy controls 2) Asthmatic controls and 3) Asthmatics following provocation.^a^

Variable	Healthy controls (HC)		Asthmatic controls (AC)		Asthmatics following provocation (AFP)		Significance^b^			
							HC/AC	HC/AFP	AC/AFP	Trend^c^
	AV^d^	CV^e^	AV	CV	AV	CV	p	p	p	p
Age	25	17	30	25	30	25	0.15	0.17	0.35	N.D.^f^
Mast cells	1.6	188	5.5	87	7.8	86	0.056	*0.023*	0.36	*0.0050*
Eosinophils	0.29	82	0.30	107	1.0	107	0.94	0.10	0.10	0.40
Neutrophils	1.3	49	0.60	73	1.2	73	*0.017*	0.66	0.093	N.D.
CD3+CD4+FOXP3	6.9	51	9.5	79	15	51	0.39	*0.016*	*0.032*	*0.0070*
CD3+CD45RO+	15	57	15	126	16	120	1.0	0.91	0.93	0.20
CD3+CD4+CXCR3+CCR4+	0.32	145	0.057	265	0.0	0.0	0.23	N.D.	N.D.	N.D.
CD3+CD4+CD161+	10	63	6.4	119	8.1	70	0.32	0.50	0.71	0.43
CD3+CD8+CD161+	10	101	2.9	87	7.6	154	0.11	0.67	0.24	0.087
CD3+CD11a+	73	38	47	57	49	29	0.11	0.068	0.80	0.32
CD3-CXCR3+	9.1	125	5.6	135	3.2	60	0.54	0.26	0.37	*0.036*
CD3-CD16+CD56+	0.88	29	0.70	130	0.5	68	0.63	0.056	0.59	0.34
CD3-CD16+CD56+CXCR3+	29	36	33	88	15	28	0.76	*0.010*	0.20	0.16
CD3-CD14+HLA-DR+	73	38	47	57	49	29	0.11	0.068	0.86	*0.036*
IL-5	4.9	79	17	65	156	93	*0.019*	*0.021*	*0.031*	*0.0060*
IL-9	4.4	101	18	65	56	78	*0.014*	*0.012*	*0.028*	*0.0030*
IL-6	367	46	709	75	997	89	0.12	0.086	0.35	0.070
IL-10^g^	5.4	144	5.5	67	9.4	76	0.97	0.29	*0.043*	0.071

a Selected variables displayed in Figure 4, cell populations are defined in Methods. Units for each of the variable groups are defined as: age (years), cell populations (% of total population in BAL cells, except for mast cells, which are given as the number of cells per 10 visual fields in a Bürker chamber), immunological markers (proportion of cells expressing a set of markers among another defined cell population as defined in Tables S4 and S5), and cytokines (fg/ml BALF) as reported in Thunberg *et al.* [42].

b Statistical significance was calculated with either an unpaired or paired Student's t-test. Values with p<0.05 are shown in italics with two significant figures.

c The median levels among the healthy controls (HC) regarding each variable were used as a cut off limit for all three groups (*i.e.*, Healthy Controls, Asthmatic Controls and Asthmatics Following Provocation). All p-values regarding trend are one-sided.

d Average.

e Coefficient of variance.

f N.D. = value not determined.

g The IL-10 data consisted of a significant range in individual values, with one healthy individual possessing a value of 26.1 fg/ml, which did not pass a Q_crit_ test. However, since this individual was included in the original Thunberg *et al.* [42] paper, it was not removed from this analysis. Exclusion of this individual from the trend test gave p = 0.042 and the resulting Student's t-test values were: HC/AC p = 0.08, HC/AFP p = 0.04, and AC/AFP p = 0.04.

### Statistical methods

The oxylipin data follow a normal or near-normal distribution (Shaprio-Wilks p>0.01). Univariate statistical analysis was performed using a Student's t-test. No multiple hypothesis testing correction was performed given that only 32 compounds were present above the LOQ, which at an α = 0.05 gives on average ∼1.6 potential false positives. In addition, the primary findings of the study focus on oxylipin products of the 15-LOX pathway (n = 15), which at an α = 0.05 gives on average <1 potential false positives. Relative percent composition was analyzed by grouping the lipid metabolites according to class. Trends in the shifts of 15-LOX-derived oxylipins and in oxylipin relative composition were evaluated between healthy and asthmatic controls, and asthmatics following allergen provocation using the Cochran-Armitage trend test [49]. The median value among the healthy controls for each oxylipin (pM) or fatty acid relative class (%) was used as a cut off limit for all three groups and all trend-based p values are one-sided. Multivariate analyses using principal component analysis (PCA) and orthogonal projections to latent structures (OPLS) were performed using SIMCA-P+ 12 (Umetrics, Umeå, Sweden) following log transformation, mean centering and UV-scaling [50]. Model performance was reported as cumulative correlation coefficients for the model (R^2^Y[cum]) and predictive performance based on seven-fold cross validation calculations (Q^2^[cum]).

## Results

### Oxylipin profiling

A total of 87 oxylipins representing 3 metabolic pathways (COX, LOX and CYP) were screened using LC-MS/MS analysis. Of these, 32 oxylipins were detected above the method LOQ. An additional 32 oxylipins were present above the method limit of detection (LOD). A complete list of screened oxylipins as well as the 32 oxylipins over the LOD, but under the LOQ, is provided in Table S1. The oxylipin levels in the BALF (individuals from all 3 groups) ranged over 4 orders of magnitude from ∼0.1 pM to 3 nM (Table S2). The average concentrations (pM) and the coefficients of variance (CV) are given for the 3 different test groups in Table 2 (*i.e.*, Healthy Controls [HC], Asthmatic Controls [AC] and Asthmatics Following Provocation [AFP]).

### Asthmatics versus healthy controls at unprovoked baseline levels

A total of 9 oxylipins were significantly elevated in the asthmatics at unprovoked baseline levels relative to healthy controls (∼2-fold increase comparing the group averages in Table 2; p≤0.05). These oxylipins were 12- and 15-HETE, epoxyketooctadecenoic acid (EKODE), 9,12,13-trihydroxyoctadecenoic acid (9,12,13-TriHOME), 15-hydroxyeicosatrienoic acid (15-HETrE), 12(13)-epoxyoctadecadienoic acid (12(13)-EpODE), 12- and 15-hydroxyeicosapentaenoic acid (12- and 15-HEPE) and 17-hydroxydocosahexaenoic acid (17-HDoHE). With the exception of EKODE and 12(13)-EpODE, all other significantly elevated oxylipins were most likely 15-LOX metabolites (Figure S2). However, it is possible that P450 activity could account for biosynthesis of some of the mono-hydroxy analogs [51].

### Asthmatics following provocation

Following birch pollen provocation, 17 oxylipins were significantly higher in the asthmatics relative to healthy controls (∼1.5- to 3-fold increase based upon the group averages in Table 2; p≤0.05). The majority of the significantly altered oxylipins (12 out of 17) were 15-LOX metabolites (Figure S2). With the exception of EKODE, all of the oxylipins elevated in the asthmatic controls were further elevated following provocation. In addition, significantly higher concentrations were observed for 15-oxo-eicosatetraenoic acid (15-KETE), 5,15-dihydroxyeicosatetraenoic acid (5,15-DiHETE), 6-*trans*-LTB_4_, PGE_2_, 17-HDoHE, 13-HODE, 9,10,13-TriHOME, 9(10)-epoxyoctadecenoic acid (9(10)-EpOME), 9,10-dihydroxyoctadecenoic acid (9,10-DiHOME) and 13-hydroxyoctadecatrienoic acid (13-HOTE). When investigating intra-individual alterations in the asthmatics in response to allergen provocation, no significant changes in oxylipin concentrations were detected (Table 2). However, a significant trend in elevated 15-LOX-derived compounds following provocation was observed (see below).

### 15-LOX metabolites and trend analysis

**Figure 1:**
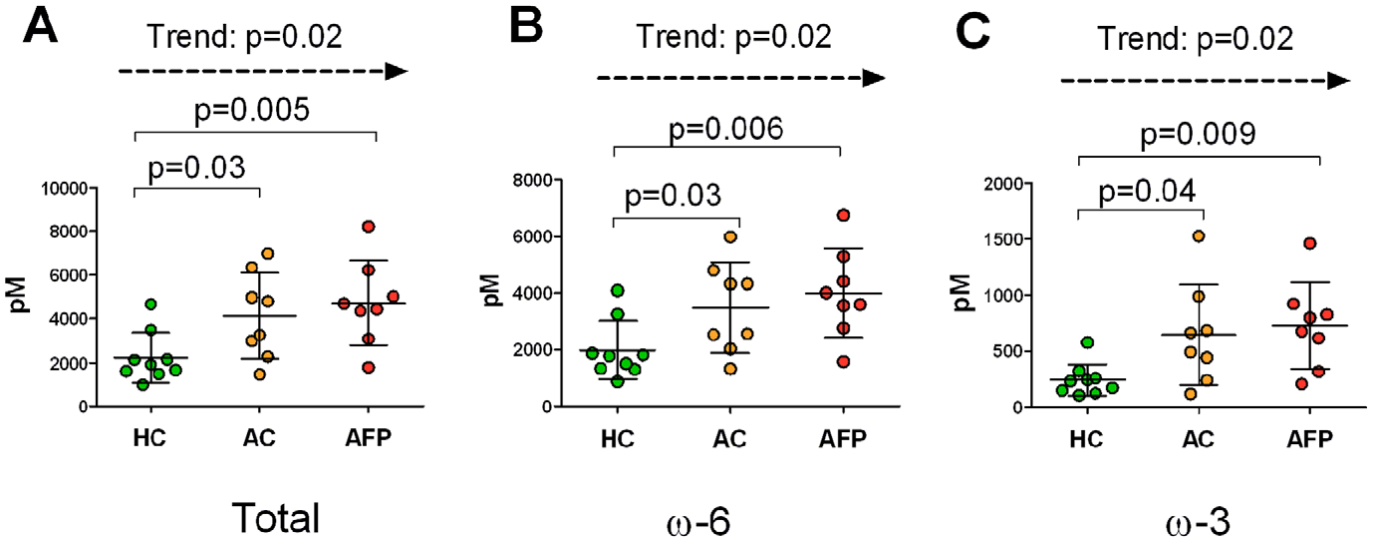
Sum of 15-LOX metabolites. (**A**) Sum of 15-LOX metabolites from both ω-3 and ω-6 pathways. (**B**) Sum of 15-LOX metabolites from ω-6 fatty acids (12-and 15-HETE, 15-KETE, 5,15-DiHETE, 13-HODE, 9,10,13- and 9,12,13-TriHOME, 9-HODE, 9-KODE and 15-HETrE), (**C**) Sum of 15-LOX metabolites from ω-3 fatty acids (9- and 13-HOTE, 12- and 15-HEPE and 17-HDoHE). Data are provided as concentration in BALF (pM). HC: Healthy controls, AC: Asthmatic controls, AFP: Asthmatics following provocation. The p-values obtained using Student's T-test (HC vs. AC and HC vs. AFP) and one-sided Cochran-Armitage trend test (HC, AC and AFP) are indicated in the figure.

Based upon the observed shifts in oxylipin levels described above, 15-LOX products were examined in further detail, both in terms of the sum of all potential 15-LOX metabolites (n = 15), as well as based upon their parent polyunsaturated fatty acid substrate divided as ω-6 (n = 10) and ω-3 (n = 5) fatty acids (Table 2, Figure 1). The sum of 15-LOX metabolites as well as the ω-6 and ω-3 grouped 15-LOX metabolites were all significantly elevated in asthmatics at baseline levels (p = 0.03, p = 0.03 and p = 0.04, respectively). The significance further increased between healthy controls and asthmatics following provocation (p = 0.005, p = 0.006 and p = 0.009, respectively). The corresponding group comparison of allergic asthmatics before and after provocation did not reach significance; however, a one-sided Cochran-Armitage trend test confirmed that the increasing oxylipin levels between healthy controls, asthmatic controls and asthmatics following provocation evidenced a trend (Table 2). The trend was significant both for the total sum of 15-LOX metabolites (p = 0.02), and for the ω-6 (p = 0.02) and ω-3 (p = 0.02) compounds compared separately (Figure 1). Further examination of the individual oxylipins revealed that the ω-6 mediators 12-HETE, 5,15-DiHETE, 13-HODE, 9,10,13- and 9,12,13-TriHOME as well as the ω-3 mediators 13-HOTE, 12-HEPE and 17-HDoHE followed the same significant trend (Table 2, Figure S2). The trend was particularly prominent for 12-HEPE (p = 0.003) and 17-HDoHE (p = 0.003). The trend was not significant for the primary 15-LOX products 15-HETE (p = 0.091) and 15-HEPE (p = 0.091).

Examination of the trend significance for compounds outside of the 15-LOX pathway evidenced additional shifts. The leukotrienes (LTB_4_ and 6-*trans*-LTB_4_) and prostanoids (PGD_2_ and PGE_2_) as well as a number of CYP products (11,12-DiHETrE, 9,10-DiHOME and 12[13]-EpODE) evidenced significant trends, increasing in levels from healthy individuals to baseline asthmatics and then asthmatics following provocation (Table 2). The trend was also examined for the cytokine and BAL cell data, with IL-5, IL-9, mast cells and T regulatory cells (Treg) CD3+CD4+FOXP3 all evidencing trends that significantly increased from healthy controls to provoked asthmatics (Table 3). IL-10 data consisted of a significant range in individual values, with one healthy individual possessing a value of 26.1 fg/ml, which did not pass a Q_crit_ test. However, since this individual was included in the original Thunberg *et al.* [42] paper, it was not removed from this analysis. Exclusion of this individual from the trend test gave p = 0.042.

### Relative fatty acid class composition

To compare global shifts in lipid metabolites, oxylipin levels were summed on the basis of their unsaturated fatty acid substrate (*e.g.*, AA). For example, all quantified oxylipins derived from AA were summed on a concentration basis and presented as percent of total quantified oxylipins. The average for the populations evidenced that BALF was highly enriched in linoleic acid (LA) metabolites, representing ∼70–80% of the overall oxylipin content as compared to ∼10–20% of AA metabolites. Other fatty acid classes (dihomo-γ-linolenic acid [DGLA], α-linolenic acid [α-LA], docosahexaenoic acid [DHA] and eicosapentaenoic acid [EPA]) only consisted of a few oxylipins relative to LA- and AA-derived metabolites, which were the dominant compounds represented in the oxylipin analytical method.

**Figure 2:**
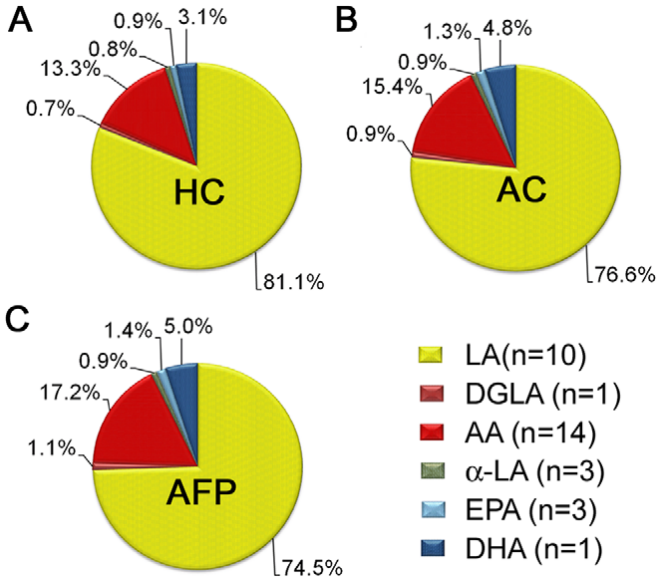
Oxylipin composition based on polyunsaturated fatty acid class. (**A**) Healthy controls (HC) (**B**) Asthmatic controls (AC) and (**C**) Asthmatics following provocation (AFP). Oxylipins were grouped into the following classes based upon their fatty acid substrate: linoleic acid (LA), dihomo-γ-linolenic acid (DGLA), arachidonic acid (AA), α-linolenic acid (α-LA), eicosapentaenoic acid (EPA) and docosahexaenoic acid (DHA). Healthy controls and asthmatics following provocation evidenced significant differences for LA (p = 0.04), EPA (p = 0.02) and DHA (p = 0.04). The proportion of EPA metabolites was also significantly higher in asthmatic controls compared to healthy controls (p = 0.04).

A comparison of the composition between the healthy and asthmatic controls only showed significant differences in the EPA metabolites (n = 3), which contributed a significantly larger proportion (p = 0.04) to the overall asthmatic oxylipin profile (1.3%) than the corresponding comparison of the pollen-exposed asthmatics relative to healthy controls (0.9%; Figure 2A and Figure 2B, Table S6). The relative composition of linoleates (n = 10) constituted a significantly smaller proportion (p = 0.04) in the profiles of pollen-exposed asthmatics (74.5%) compared to healthy controls (81.1%), and a significant higher proportion (p = 0.02 and p = 0.04, respectively) of the eicosapentaenoates (n = 3; 1.4% compared to 0.9%, respectively) and the docosahexaenoate (n = 1; 5.0% compared to 3.1%, respectively; Figure 2A and Figure 2C, Table S6). No significant alterations in fatty acid class composition were detected comparing asthmatic control profiles to the profiles following provocation. With the exception of DHA (17-HDoHE; p = 0.03), the Cochran-Armitage trend test did not indicate any significant shifts comparing healthy controls, asthmatic controls and asthmatics following provocation (data not shown).

Oxylipin levels of LA and AA origin were also summed on the basis of functional group or molecular class (*e.g.*, alcohols, ketones, epoxides and diols). The main difference in distribution was observed in the epoxides, LA (∼60%) compared to AA (∼1%; Figure S3). No significant differences were detected between healthy controls and the asthmatic groups.

### Integrated multivariate modeling

**Figure 3:**
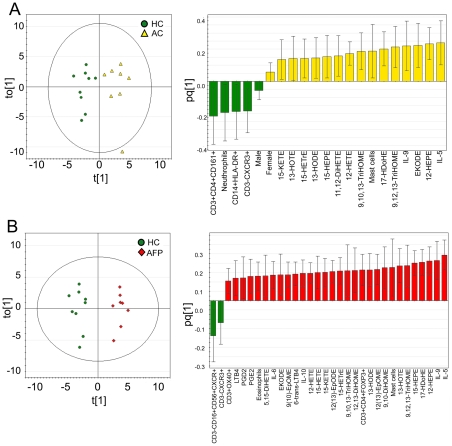
OPLS score and loading column plots with respect to separation according to diagnosis. Loading column plots visualize variables correlating with healthy (−) or asthmatics (+), error bars indicate 95% confidence interval. The number of variables correlating with the asthmatic population with 95% confidence increases following provocation from n = 21 to n = 32. (**A**) Healthy controls (green) and asthmatic controls (yellow) (R^2^Y[cum] = 0.87, Q^2^[cum] = 0.51). (**B**) Healthy controls (green) and asthmatics following provocation (red) (R^2^Y[cum] = 0.95, Q^2^[cum] = 0.73).

Multivariate statistical modeling integrating oxylipin data with previously published clinical information (FACS characterizations of BAL cells, cytokine and differential inflammatory cell data [42]) was performed in order to investigate the utility and biological relevance of oxylipin levels in the light of established inflammatory markers. OPLS analysis with respect to separation according to diagnosis was performed for A) Healthy Controls and Asthmatic Controls (Figure 3A) and B) Healthy Controls and Asthmatics Following Provocation (Figure 3B). The resulting models were termed “Baseline Asthmatics” and “Provoked Asthmatics”, respectively. OPLS is a supervised multivariate method that allows extraction of the variance of interest (here the difference between asthmatics and healthy) from the unrelated (orthogonal) noise in the data set. This separation of the predictive variance from the orthogonal variance greatly improves the interpretability of the resulting model, because the contribution of the specific original data variables (*e.g.*, oxylipins) can easily be derived from the loadings of the single predictive vector [50]. Both OPLS models were constructed from one predictive and one orthogonal component, with both resulting in a good separation between healthy controls and asthmatics (Baseline Asthmatics: R^2^Y[cum] = 0.87, Provoked Asthmatics: R^2^Y[cum] = 0.95). Seven-fold cross validation revealed that the models were robust (Baseline Asthmatics: Q^2^[cum] = 0.51, cross-validated analysis of variance (CV-ANOVA) = 0.15; Provoked Asthmatics: Q^2^[cum] = 0.73, CV-ANOVA = 0.01 ), corresponding to 51% predictive power at unprovoked baseline levels, and 73% predictive power following allergen provocation. The subject located outside of Hotelling's T^2^ in the Baseline Asthmatics model (Subject 1, Figure 3A) was an outlier only in terms of the orthogonal vector, and further examination of the Distance to Model X (DModX) confirmed that this individual was within the 95% confidence interval. As such, the potential outlier was not removed from further analyses. The loading column plots (Figure 3; A) n = 21; B) n = 32) show that predominantly the cytokine and oxylipin variables drive the separation between groups.

Comparison between the two models (Baseline Asthmatics and Provoked Asthmatics) was performed using a Shared and Unique Structures (SUS) plot, in which the loadings of the predictive vectors for the two models are plotted against each other to reveal differences and similarities in the variable contribution between models [52]. In the SUS plot, the predictive vectors (p) are also scaled as a correlation coefficient (p[corr]) between the actual datum points (X-matrix) and the corresponding scores vector (t), making the plot interpretation more intuitive. A high p(corr) indicates a high prominence of the variable in driving the separation between groups in the particular model. For example, the relatively higher p(corr) for IL-10 in the Provoked Asthmatics model (0.5), compared to the Baseline Asthmatics model (0.1), indicates that IL-10 is more important for driving the separation between healthy and asthmatics following allergen provocation than at baseline levels. The SUS plot can be interpreted based upon variable location in the plot, with shared variables located on the diagonal (either positive or negative) and unique effects for Baseline Asthmatics and Provoked Asthmatics found closer to the X- or Y-axis, respectively.

**Figure 4:**
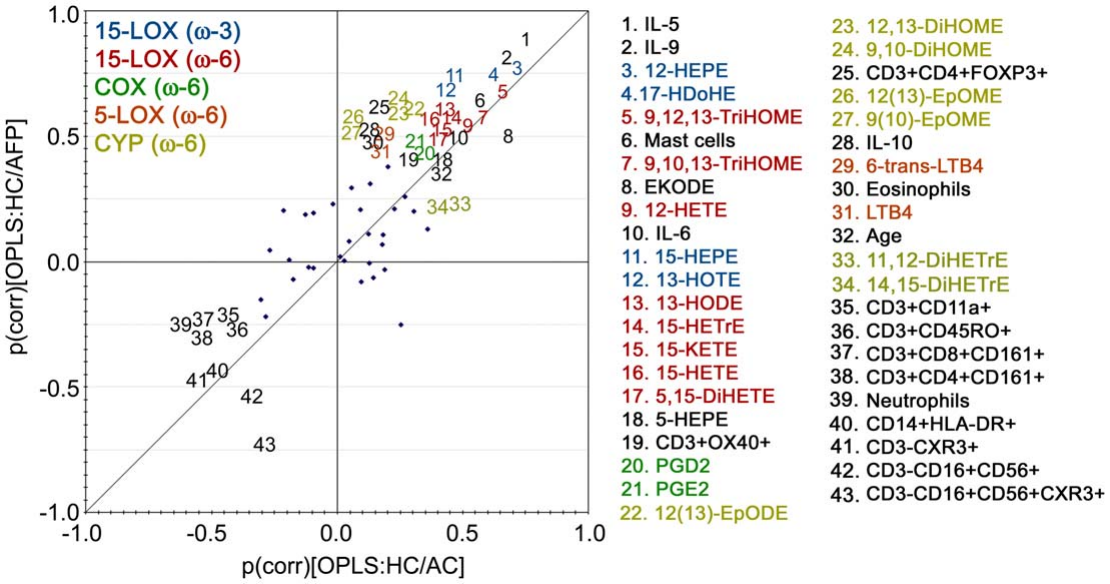
Shared and Unique Structures (SUS) plot. SUS plot correlating the OPLS models of healthy controls versus asthmatic controls (Baseline Asthmatics, Figure 3A, X-axis) and healthy controls versus asthmatics following provocation (Provoked Asthmatics, Figure 3B, Y-axis). A complete list of p(corr) values for both models is provided in Table S7. Abbreviations are as follows: 15-lipoxygenease (15-LOX), cyclooxygenase (COX), 5-lipoxygenase (5-LOX), cytochrome P450 (CYP), ω-6 fatty acid (ω-6), ω-3 fatty acid (ω-3), healthy controls (HC), asthmatic controls (AC) and asthmatics following provocation (AFP). Colors are as follows: 15-LOX-derived ω-3 oxylipins (blue), 15-LOX-derived ω-6 oxylipins (red), COX-derived ω-6 oxylipins (green), 5-LOX-derived ω-6 oxylipins (orange), CYP-derived ω-6 oxylipins (gold). Variables with p(corr)≥|0.4| are labeled, while those ≤|0.4| are shown as symbols.

The SUS plot demonstrated a strong positive correlation of the variable contribution between the two models (R^2^ = 0.7, Figure 4). The primary shared structures were the 15-LOX-derived ω-6 and ω-3 metabolites, the cytokines IL-5 and IL-9 as well as the mast cells, which all showed robust positive correlation with the asthmatic individuals in both models (Figure 4). However, the 15-LOX ω-3 oxylipins had a slightly stronger contribution to the Provoked Asthmatics model. In particular, the p(corr) values for both 15-HEPE and 13-HOTE were 0.4 and 0.7 for the Baseline Asthmatics and Provoked Asthmatics models, respectively (Figure 4, Table S7). In contrast, the 15-LOX ω-6-derived oxylipins evidenced little variation between the two models, with 15-HETE, 15-KETE, 15-HETrE and 13-HODE possessing similar p(corr) values (p[corr] = 0.4 and 0.6 for the Baseline Asthmatics and Provoked Asthmatics, respectively; Figure 4, Table S7). The COX-derived ω-6 oxylipins PGE_2_ and PGD_2_ co-localized in the SUS plot, with little contribution to the separation between groups. IL-5 and IL-9 had the strongest contributions to driving the overall models with p(corr) values ranging from 0.7–0.9 for both Baseline and Provoked Asthmatics. Mast cell abundance had a weaker, but identical, contribution to both Baseline and Provoked asthmatics (p[corr] = 0.6).

The primary unique structures were the CYP-derived linoleic acid products, which were more prominent variables in the Provoked Asthmatics model. The strongest contributions were from the epoxides 9(10)-EpOME and 12(13)-EpOME, which shifted from no contribution at Baseline levels (p[corr] = 0.0–0.1) to 0.5 and 0.6 respectively for the Provoked Asthmatics model (Table S7). The corresponding diols, 9,10-DiHOME and 12,13-DiHOME, evidenced a similar shift, but with slightly stronger contributions at Baseline levels (p[corr] values for both species shifted from 0.2 to 0.6). The 5-LOX-derived ω-6 oxylipins LTB_4_ and 6-*trans*-LTB_4_ were also unique for the Provoked Asthmatics model, but with slightly weaker contributions (p[corr] = 0.4–0.5). The only oxylipin variables to contribute stronger to the Baseline Asthmatics models were the ω-6 AA diols 11,12-DiHETrE and 14,15-DiHETrE, which exhibited p(corr) values of 0.2 for Provoked Asthmatics relative to 0.5 and 0.4 for Baseline Asthmatics, respectively (Figure 4, Table S7). In terms of the cytokines and inflammatory cells, the unique variables for the Provoked Asthmatics were IL-10 and eosinophil abundance, which both possessed p(corr) values of 0.5 relative to 0.1 for Baseline Asthmatics (Figure 4, Table S7). In addition, CD3+CD4+FOXP3+ Treg cells contributed stronger to the Provoked Asthmatics model with a p(corr) of 0.6 relative to 0.2 for Baseline Asthmatics (Figure 4, Table S7). CD3-CD16+CD56+CXCR3+ cells evidenced an inverse contribution to the Provoked Asthmatics model (p[corr] = −0.7).

## Discussion

One of the primary observations in this study was that asthmatic controls demonstrated a distinct lipid mediator profile relative to healthy controls. These individuals are mild intermittent asthmatics that are essentially “healthy” at baseline when no allergen provocation has occurred for at least 3 weeks. However, the same pattern of elevation in 15-LOX metabolites observed following allergen provocation was present at baseline levels. Of the altered 15-LOX-derived compounds, AA-derived 15-HETE is the most well known indicator of pro-inflammatory responses in asthma [31], [32]. It is likely that the significantly elevated levels of 15-HETE in baseline asthmatics signify pro-inflammatory processes active in stabile, unprovoked mild asthmatics, which are further amplified following allergen provocation.

The ratio of 15-HETE levels in BALF for baseline asthmatics vs. healthy individuals was 2∶1, which agreed with previous studies [32], [47]. A comparison of all 15-LOX products from AA-derived oxylipins gave a ratio of ≥2 between asthmatics and healthy individuals, whereas for LA-derived oxylipins the ratios were ≤2 (in agreement with earlier results [47]). The ratios of 15-LOX products from ω-3 fatty acids were all ≥2 between asthmatics and healthy individuals, which differs from our earlier studies of mild intermittent asthmatics [47]. This observation may reflect higher variability in ω-3-derived oxylipins, which could in part be a function of their lower physiological levels. The biological significance of the differing ratios is unclear, but becomes more interesting when examined in terms of BALF composition, of which LA-derived oxylipins are the dominant species (Figure 2). For example, in the global oxylipin profile based on relative fatty acid mediator composition (%), the ratios of LA∶AA metabolites were similar for healthy and asthmatic controls (6.6 and 5.9, respectively compared to 5.2 for allergen provoked asthmatics). However, we have previously observed significant differences in BALF oxylipin LA∶AA ratios of healthy (LA∶AA = 6.3) and baseline asthmatics (LA∶AA = 3.4) [47]. These observations suggest that biological processes related to AA-derived oxylipins play a more prominent role in asthmatics relative to LA-derived mediators. These differences potentially reflect shifts in 15-LOX activity between the different groups and given the biological significant of these oxylipin mediators, warrant further investigation.

Some of the individual oxylipins quantified in this study have been previously examined in pulmonary disease (see Lundström *et al.* for a comprehensive review [24]), but there is only one known previous study performing oxylipin metabolic profiling in BALF from asthmatics [47] and no known studies from other inflammatory conditions (*e.g.*, COPD, hypersensitivity pneumonitis or sarcoidosis). To place our findings within the context of the known literature, we have examined a few of the well-described compounds in terms of their contribution to the multivariate modeling. Prostanoids are potent mediators of inflammation in asthma that evidenced Shared (equal) contributions to the Baseline and Provoked Asthmatics models in the SUS plot. The biological role of PGD_2_ and PGE_2_ in allergic inflammation is multifaceted, with PGD_2_ causing bronchoconstriction, elevating Th2 cytokines [53] and eosinophil infiltration into the lung; roles that are consistent with the location of PGD_2_ in the SUS plot. PGE_2_ on the other hand prevents allergen-induced bronchoconstriction [21], decreases airway eosinophilia [54] and activates mast cells [55]. The proximity of PGE_2_ in the SUS plot to eosinophils and IL-10 potentially suggests ongoing suppression of eosinophilia, which would be expected at 24 h post-allergen exposure. The multivariate modeling shows that the pro-inflammatory cytokines in combination with the anti-inflammatory oxylipins contribute strongly and similarly to the models in both Baseline and Provoked Asthmatics at 24 h, suggesting that these mediators do not play a significant role in allergen-specific processes. While the trend is significant, the prostanoids have an overall weaker contribution to the model. Accordingly, it is possible that these variables are a reflection of the overall inflammatory state in these asthmatics, which is further amplified following allergen-provocation; however, no new pathways are affected during this process. This interpretation is supported by the significant trend of increasing levels for the majority of the quantified 15-LOX-derived oxylipins.

The multivariate model was also interrogated for allergen-specific processes, with the Provoked Asthmatics model illustrating a similar mix of pro- and anti-inflammatory markers. In this phase, eosinophils significantly increased, most likely accounting for the corresponding increase in leukotrienes (*e.g.*, 6-*trans*-LTB4). However, eosinophils are suppressed by IL-10 [56], which also increased following allergen provocation. The eosinophils and the LA-derived CYP-produced EpOMEs and DiHOMEs shifted from essentially no contribution (p[corr]≤0.1) to the principle unique driving variables in the Provoked Asthmatics model (p[corr]≥0.6; Figure 4). This shift in the SUS plot evidences an effect on the atopic asthmatic lung from birch pollen that was not observed in unprovoked asthmatics. Extreme levels of EpOMEs and DiHOMEs are associated with acute respiratory distress syndrome (ARDS), in which high levels are detected (*e.g.*, 38.5±21.9 nM for 9[10]-EpOME) [57], [58]. In the current study, levels were much lower, but still evidenced a significant increase in asthmatics following provocation (1.6±0.4 nM relative to 0.9±0.3 nM for healthy controls). In contrast, the increase in CD3+CD4+FOXP3+ Treg cells following allergen provocation may be indicative of an attempt to launch an anti-inflammatory response in these mildly asthmatic subjects (Figure 4). However, Hartl *et al.* [59] showed that asthmatic children were associated with a decreased number of Treg cells, which were also shown to possess an impaired functionality, rendering the subject with an inability to regulate the allergen-induced inflammation. Likewise, *in vitro* stimulation studies performed by Thunberg *et al.* [42] on primary peripheral blood mononuclear cells (PBMCs) from the same subjects in this study showed an inability to suppress Th2 responses, indicating that the infiltrating FOXP3+ cells are not able to control the allergen-induced Th2 response [42]. The enhanced levels of pro-inflammatory oxylipins such as the 15-LOX-derived ω-6 oxylipins and leukotrienes provide further support of these conclusions.

This study focused on allergen-provoked asthmatics at the 24 h time point. Allergen inhalation by allergic asthmatics has two distinct temporal components: 1) The first event is the allergen-induced early asthmatic response (or the immediate IgE-mediated reaction), which involves an acute bronchoconstrictor response that develops within 15 min and usually resolves within 2 h and 2) the second period of bronchoconstriction or the late asthmatic response beginning 3–4 h after inhalation, often peaking at 6–9 h, and lasting up to 24 h [8], [22]. The late-phase reaction often consists of Th2 cells, which affects the cytokine environment and eosinophil levels [60], [61]. Accordingly, the snapshot of mediators acquired in this study provides an image of the allergic response as it shifts towards a resolution phase. While no oxylipins known to be directly involved in this phase (*e.g.*, lipoxins, resolvins or protectins [17]) were observed, a number of ω-3 products including the 15-LOX-derived 17-HDoHE were increased at 24 h. 17-HDoHE is derived from the 17-HPDoHE peroxide precursor, which can alternatively be converted to the 17-D series of resolvins (17-RvDs) [17]. These observations in combination with the IL-10 and CD3+CD4+FOXP3+ data are suggestive of a shift towards an anti-inflammatory milieu at this stage.

Taken together these results indicate that 1) even mild allergic asthmatics have altered oxylipin profiles relative to healthy controls, 2) these differences are further augmented by allergen-provocation suggesting that the profiles are indicative of background asthma-based pathology at baseline and 3) by 24 h post-allergen exposure, mild allergic asthmatics exhibit a mixed pro- and anti-inflammatory phenotype that is most likely indicative of an ongoing resolution process. It is also possible that oxylipins are more long-lived and subsequently the late asthmatic phase is longer than expected, which was not observed with other inflammatory markers. This theory would require confirmation via time course-based studies designed to quantify oxylipin half-life throughout the inflammatory process.

### Conclusion

The oxylipin profile of mild allergic asthmatics is distinct from that of the healthy lung at baseline and these differences are amplified following birch allergen provocation. The predominant shifts were in the 15-LOX pathway, further stressing the role of this pathway in asthma pathology. The simultaneous elevation of both pro- and anti-inflammatory oxylipins was highlighted by multivariate analyses, in which cytokine levels in combination with inflammatory cell data also correlated with asthmatics both at baseline levels and following provocation. The observation that allergen exposure amplified an existing underlying difference between healthy subjects and baseline asthmatics indicates the presence of a basal level of inflammation. These asthmatics were non-symptomatic, stable subjects with mild intermittent asthma, yet still evidenced a distinct quantifiable inflammatory signature associated with disease pathology that was not discernable by traditional inflammatory mediators (with the exception of a slight elevation of IL-5 in BALF [42]). Accordingly, these results provide further support for the use of oxylipin profiling as a more nuanced and sensitive means of detecting underlying low levels of inflammation in asthmatics and potentially other respiratory disorders. In addition, the significant shifts in the oxylipin signature between the baseline asthmatics and healthy controls suggests that even non-symptomatic individuals possess unique disease-specific phenotypes/metabotypes that have clinical ramifications as well as provide insight into the etiology and pathology of disease.

## Supporting Information

Figure S1 **Sample chromatograms from the oxylipin metabolic profiling method.** **A**) Full chromatogram from asthmatic individual 7 in Table 1 following provocation showing the range of oxylipins detected. **B**) Extracted ion chromatograms for selected compounds (PGD_2_ and PGE_2_; 6-*trans*-LTB_4_, LTB_4_, and purported isomers; 5,6-DiHETE and purported isomers). Peaks shaded black indicate integrated peaks, while non-shaded peaks possess the same mass transition and represent potential isomers; however, are not reported due to a lack of analytical standards.
(TIF)

Figure S2 **Oxylipins derived from the 15-LOX pathway.** **A**) Oxylipins derived from ω-6 fatty acids (AA, DGLA, and LA). **B**) Oxylipins derived from ω-3 fatty acids (α-LA, EPA, and DHA). HC: healthy controls, AC: asthmatic controls, AFP: asthmatics following provocation. Numbers shown in each graph are the p-values obtained using a Student's T-test (HC vs AC and HC vs AFP, respectively). The p-values for the one-sided Cochran-Armitage trend test (HC, AC and AFP) are indicated below each graph.
(TIF)

Figure S3 **Oxylipin class profiles for linoleic acid- (LA) and arachidonic acid- (AA) derived compounds.** No significant differences were detected between healthy controls and asthmatics. Alcohols: LA (13-HODE and 9-HODE), AA (5-HETE, 12-HETE and 15-HETE). Ketones: LA (9-KODE), AA (15-KETE). Epoxides: LA (9(10)-EpOME and 12(13)-EpOME), AA (11(12)-EpETrE). Diols: LA (9,10-DiHOME and 12,13-DiHOME) and AA (LTB_4_, 6-*trans*-LTB_4_, 5,15-DiHETE, 11,12-DiHETrE, 14,15-DiHETrE and 5,6-DiHETE). An oxylipin nomenclature list is provided in Table S1.
(TIF)

Table S1 **Standard list, nomenclature and information on detected and quantified oxylipins in the study.** Standards include analytical-, deuterated internal- and technical standards. Yellow markings indicate if oxylipins were assigned over the limit of detection (LOD) or limit of quantification (LOQ).
(XLSX)

Table S2 **Absolute concentrations of quantified oxylipins not adjusted to bronchoalveolar lavage (BAL) recovery volumes.** Concentrations are given in pM.
(XLSX)

Table S3 **Bronchoalveolar lavage (BAL) recoveries of instilled and recovered volume in participating subjects.** Volumes are given in mL.
(XLSX)

Table S4 **FACS panels.** A list of the fluorophor conjugated antibodies to the listed surface markers (Except FOXP3, which is intracellular).
(XLSX)

Table S5 **FACS cell populations.** The quantified cell populations among lymphocytes or total BAL cells are defined by the FACS gating strategy as percentage within defined gates, as shown within parentheses.
(XLSX)

Table S6 **Relative percent composition of quantified oxylipins on an individual and fatty acid class-specific basis.**
(XLSX)

Table S7 **List of p(corr) values from the OPLS models.**
(XLSX)

Table S8 **Oxylipin concentrations in BALF from 1) Healthy controls 2) Asthmatic controls and 3) Asthmatics following provocation provided in units of median with min/max values.** Concentrations are given in pM.
(XLSX)
