# Supplementary figures and images for: Allergic Asthmatics Show Divergent Lipid Mediator Profiles from Healthy Controls Both at Baseline and following Birch Pollen Provocation
Source: PLoS One. 2012 Mar 15;7(3):e33780. doi: 10.1371/journal.pone.0033780 (PMC3305349; doi:10.1371/journal.pone.0033780)

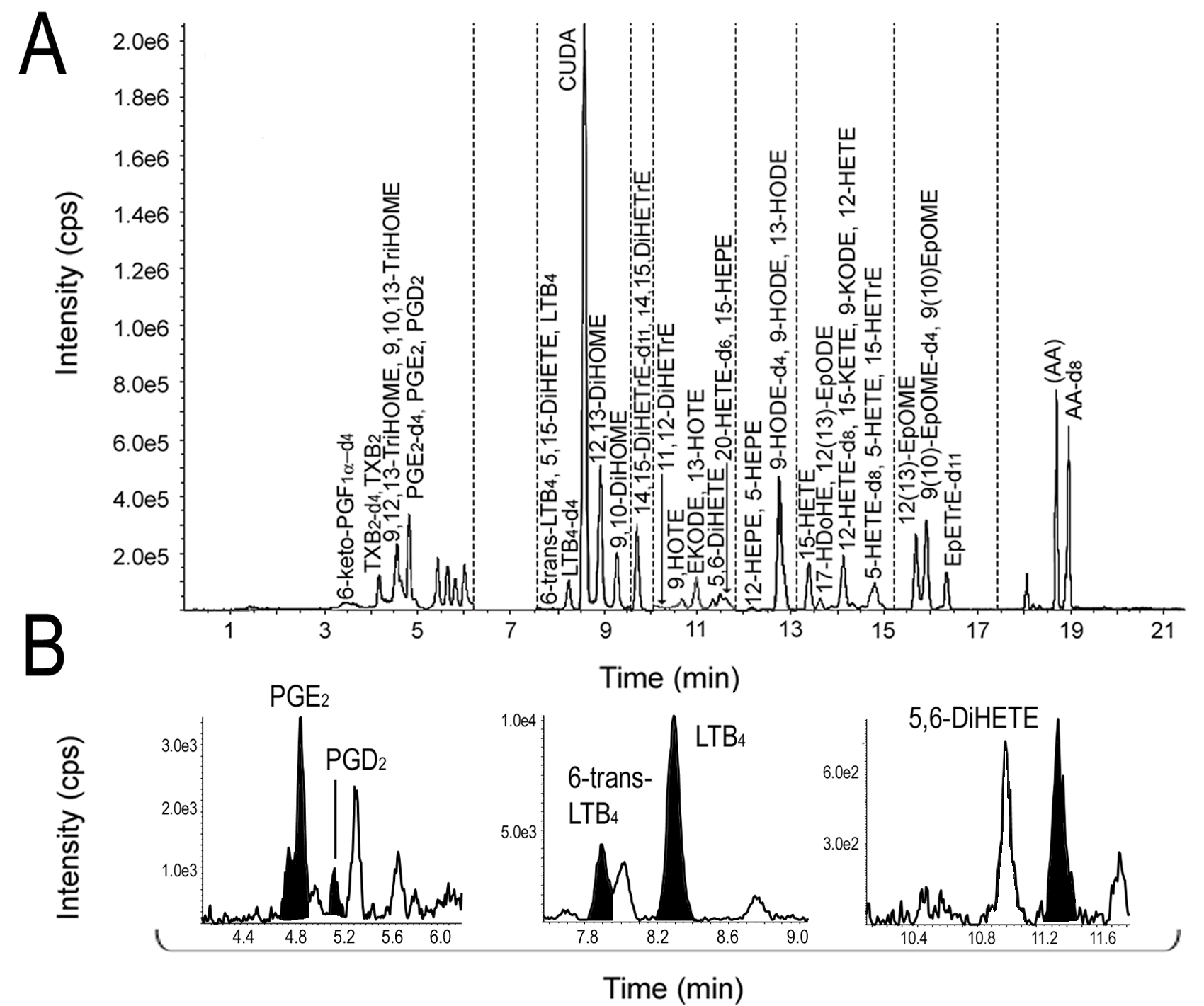

Supplement: Figure S1 — Sample chromatograms from the oxylipin metabolic profiling method. A) Full chromatogram from asthmatic individual 7 in Table 1 following provocation showing the range of oxylipins detected. B) Extracted ion chromatograms for selected compounds (PGD2 and PGE2; 6-trans-LTB4, LTB4, and purported isomers; 5,6-DiHETE and purported isomers). Peaks shaded black indicate integrated peaks, while non-shaded peaks possess the same mass transition and represent potential isomers; however, are not reported due to a lack of analytical standards. (TIF) [file pone.0033780.s001.tif]
